# *De novo* backbone scaffolds for protein design

**DOI:** 10.1002/prot.22651

**Published:** 2009-11-05

**Authors:** James T MacDonald, Katarzyna Maksimiak, Michael I Sadowski, William R Taylor

**Affiliations:** Division of Mathematical Biology, MRC National Institute for Medical Research, The RidgewayMill Hill, London NW7 1AA

**Keywords:** computational protein design, *de novo* scaffold construction, coarse-grained potential energy function, synthetic biology

## Abstract

In recent years, there have been significant advances in the field of computational protein design including the successful computational design of enzymes based on backbone scaffolds from experimentally solved structures. It is likely that large-scale sampling of protein backbone conformations will become necessary as further progress is made on more complicated systems. Removing the constraint of having to use scaffolds based on known protein backbones is a potential method of solving the problem. With this application in mind, we describe a method to systematically construct a large number of *de novo* backbone structures from idealized topological forms in a top–down hierarchical approach. The structural properties of these novel backbone scaffolds were analyzed and compared with a set of high-resolution experimental structures from the protein data bank (PDB). It was found that the Ramachandran plot distribution and relative γ- and β-turn frequencies were similar to those found in the PDB. The *de novo* scaffolds were sequence designed with RosettaDesign, and the energy distributions and amino acid compositions were comparable with the results for redesigned experimentally solved backbones. Proteins 2010. © 2009 Wiley-Liss, Inc.

## INTRODUCTION

The process of computational protein design can be split into two coupled problems. The first problem is selecting or generating a backbone scaffold that is “designable.” The second problem is to find sequences that are able to fold into the desired backbone structure. The two problems are coupled in the sense that it is unlikely that sequences exist that are able to fold an arbitrary backbone structure.^1^ The first problem can be solved most simply by taking the backbone from an experimentally solved protein as at least one sequence is known to fold into that structure. Sequence design for a fixed backbone can be approached by finding low energy sequences by a stochastic method such as Monte Carlo search^2^,^3^ or with the deterministic algorithm Dead-end elimination.^4^,^5^ The two problems can be partially recoupled by allowing some backbone flexibility during the sequence redesign step.^6^,^7^ It seems that minimizing the potential energy of a sequence for a given backbone is sufficient to produce experimentally foldable designs without considering alternative conformational states,^8^,^9^ but negative design methods have been successfully experimentally verified and may prove to be important for more complex systems.^10^,^11^

Over the past several years, the progress of computational protein design has been such that it has even been possible to engineer new functionality onto preexisting backbone scaffolds,^12^–^15^ and, in some cases, this has involved loop remodeling.^16^ Although there has also been progress in constructing novel scaffolds for *de novo* design,^7^,^17^ this remains an open problem, and ultimately one would not want to be restricted to a limited set of possible backbones.^1^ In relation to this, it is interesting to note that the experimental observation of novel protein folds is becoming rare,^18^,^19^ but the number of possible single domain topologies that have not yet been seen is proposed to be an order of magnitude greater.^20^

The two successful *de novo* backbone construction strategies have been to construct backbones from fragments of known proteins with imposed distance restraints^7^ or to take a hierarchical approach and build up from idealized segments of secondary structure.^17^,^21^ In this article, we present a novel method to construct *de novo* backbone scaffolds using a hierarchical strategy with simple geometric rules together with a coarse grained potential energy function.

A hierarchical scheme to classify protein topology in a “Periodic Table”^22^ has previously been applied to protein structure prediction with particular emphasis on larger folds that are difficult to solve with existing *de novo* methods.^23^ The “Periodic Table” classifies compact globular protein domains into layers of secondary structure imposed by β-sheets. Ideal forms, in which each secondary structure element is represented as a line segment, are generated with simple packing rules.^22^ The axes of two packing α-helices or an α-helix packing on a β-sheet are placed 10 Å apart, whereas adjacent strands in a β-sheet are placed 5 Å apart. The β-sheets have a predefined “twist,” “curl,” and “stagger,” which the packing α-helices follow. Connections between the secondary structure elements in the layers then define a topology resulting in a “stick” model.^24^ It is then possible to construct rough α-carbon structures using the sticks as axes for placing idealized α-carbon secondary structure elements.^25^

With an emphasis on providing possible novel scaffolds for protein design, we present a method to construct protein backbone structures directly from these ideal forms and assess their quality with an analysis of their local backbone conformations. Sequences for these decoy backbones were designed and relaxed with Rosetta^26^ together with a set of real protein backbones and a set of compact random walk backbones as controls. The Rosetta and dDFIRE^27^ potential energy functions were used as heuristics to assess the “designability” of the decoy backbones in comparison with real backbone controls. As part of the method to produce protein-like backbones, we also present a novel structural alphabet-based α-carbon homopolymer potential energy function that was mainly parameterized to provide protein-like local structural properties and good hydrogen bonding.

## METHODS

**Figure 1:**
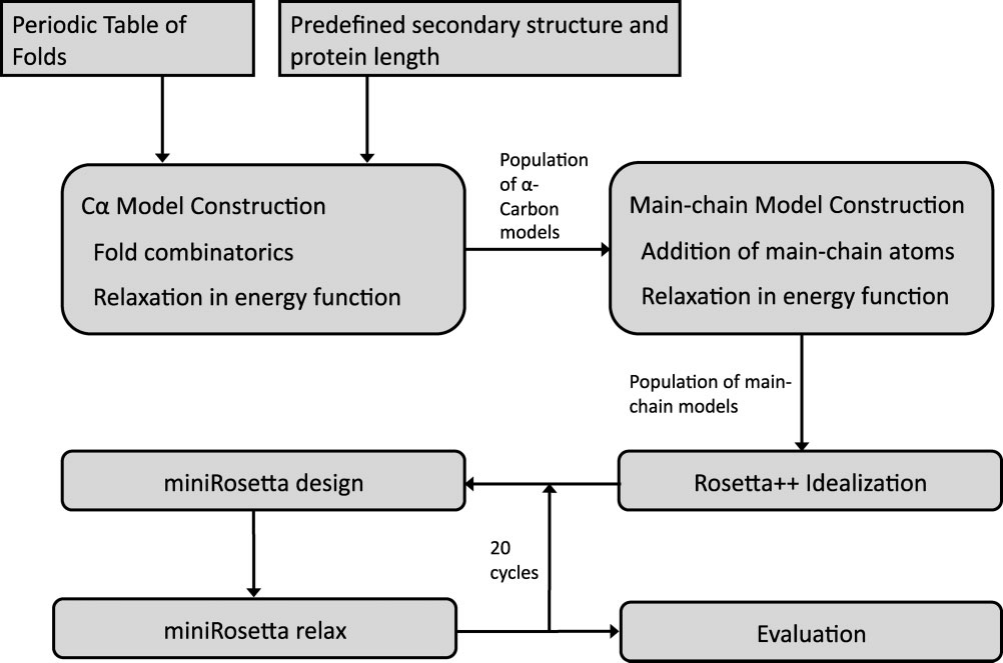
Scaffold construction and design protocol.

See Figure 1 for an overview of the method.

The method extends and refines methods previously developed within the group.^22^,^23^,^25^ Given a predefined secondary structure and a set of ideal forms (i.e. secondary structure elements arranged into layers),^22^ all possible topologies were enumerated excluding “forbidden” arrangements such as left-handed β-α-β connections and internal parallel connections. This produced “stick” models of the possible topologies where each “stick” represented the axis of a secondary structure element. An initial α-carbon model was constructed on the “sticks” using a previously described method.^25^

The backbone structure of a protein is largely defined by the positions of the α-carbons. As it is possible to quickly explore conformational space at the α-carbon level, it was decided to construct a coarse-grained homopolymer potential energy function to relax the initial α-carbon model before adding on the other main chain atoms. This potential energy function consisted of terms that represent the α-carbon-α-carbon pseudobond, pseudohydrogen bonding, a soft steric repulsive term, a radius of gyration-based term for compactness, and terms to restrict the local structure to protein-like conformations. This potential energy function differed from other previously developed coarse-grained potential energy functions in that it was designed to be used solely to provide protein-like local main-chain conformations and hydrogen bonding without any consideration given to sequence dependent properties. In contrast, other coarse potential energy functions are optimized for structure prediction or other similar applications where it is more important to get good overall tertiary structure.^28^–^31^

The α-carbon Monte Carlo move set was composed of local crankshaft moves, torsion angle rotations, bond angle rotations, bond length moves, and single atoms moves in Cartesian space. After each move, the Metropolis criterion was applied.

Having refined the initial α-carbon model, main-chain atoms (C′, O, N, and Cβ) were added using a method derived from Milik *et al*.^32^ with an additional conjugate gradient descent minimization step and 2000 steps of main-chain Monte Carlo to make small adjustments to the main-chain structure. Because no sequence is yet associated with the structure, the main chain is modeled as a simple polyalanine homopolymer at the main-chain stage.

The main-chain potential energy function consisted of a reimplementation of the Rosetta hydrogen bond potential,^33^,^34^ bonded and nonbonded interactions between atom pairs up to 1–6 from the OPLS-UA^35^ force field, the same radius of gyration term as used for the α-carbon potential energy function, and soft steric repulsion term for atom pairs over 1–6.

Main-chain Monte Carlo moves were back-rub moves,^36^ small torsion angle rotations, and bond angle rotations.

The main-chain models were idealized as poly-alanine with Rosetta++ and put through 20 cycles of design and relaxation using the miniRosetta applications fixbb and relax.

### The α-Carbon Potential Energy Function

The α-carbon potential energy function is composed of eight terms.

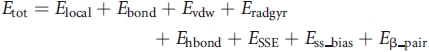
(1)
*E*_local_ is a local conformational energy and is composed of pseudobond angle and dihedral terms. *E*_bond_ is a pseudobonding term between α-carbons. *E*_vdw_ is a soft steric repulsive term. *E*_radgyr_ is a radius of gyration term to ensure the chain remains compact. *E*_hbond_ is a pseudohydrogen bonding term. The last three terms (*E*_SSE_*, E*_SS_bias_, and *E*_β_pair_) are designed to keep the secondary structure elements close to the ideal tertiary structure as defined in the ideal forms.

The core of the potential energy function was based on a 4-mer structural alphabet with 27 “letters” with each letter being associated with one torsion angle (τ) and two bond angles (θ_1_ and θ_2_) [Fig. 1(a)]. Each of these letters represented high-density states observed in the protein data bank (PDB) determined using a clustering algorithm (Pandini A, Kleinjung J. Structural alphabets derived from attractors in conformational space. 2009; submitted). Using best local RMSD fit to a letter, each 4-mer in a high-resolution training set was classified. Each 4-mer was also classified into three binned α-carbon-α-carbon distances [*d*_*i,i*+2_, *d*_*i,i*+3_, and *d*_*i*+1*,i*+3_, where *d*_*i,i*+3_ is also given a sign depending on whether the fourth α-carbon is above or below the plane defined by the first three α-carbons; Fig. 1(a)] and for each combination of these bins, *b*_combined_, the frequency of each “letter” *A* as classified by best local RMSD fit was calculated. From this information, a lookup table was created where each bin, *b*_combined_, was assigned to the letter *A* where (2) was at a maximum as a function of *A*. Bins with no counts were classified as belonging to the same letter as the nearest classified neighbor. This faster distance-based classification scheme was created to avoid more computationally costly RMSD fits.

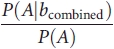
(2)

Each residue in each classified letter in the high-resolution training set had associated with it a secondary structure resulting in a 3-class secondary structure strings such as HHHH, EEEE, --EE, HH--, and ---. If the peptide bond between residues 2 and 3 in the 4-mer was in a *cis* conformation, this was classified as a “*cis*” 4-mer. If the secondary structure string was of the form HHXX or XXHH, it was classified as “helical,” and similarly if the string was of the form EEXX or XXEE, then it was classified as “strand.” All other conformations were classed as “other.” For each letter, the frequencies of each secondary structure class in the training set were recorded. The letters were then further classified as belonging to the “helical,” “strand,” “*cis,*” or “other,” where (3) was at a maximum as a function of secondary structure class.

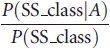
(3)

The functional form for each of the three angles in a 4-mer [Fig. 1(a)] was assumed to be a harmonic potential:

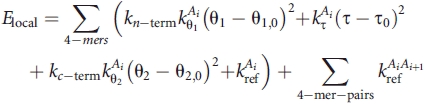
(4)
where *k*_*n*-term_ was set to 1 when the 4-mer was the N-terminal letter but was otherwise set to 0.5, and *k*_*c*-term_ was set to 1 when the 4-mer was the C-terminal letter but was otherwise set to 0.5 in order to account for overlapping 4-mers.

The equilibrium angle terms in *E*_local_ were set to the corresponding value in the structural alphabet letter, and spring constant terms for each angle/letter were related to the observed variance of the angle/letter in the training set by:

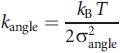
(5)

For each letter, *A*, a correctional term, *k*_ref_, was defined to ensure the same equilibrium distributions of *A* were observed as in the PDB. These reference energies were set by relaxing each of the structures in the high-resolution training set in the potential energy function for 2 million steps of Monte Carlo and setting the reference energy to:

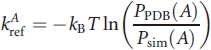
(6)
where *P*_PDB_ is the observed probability distribution in the training set and *P*_sim_ is the probability distribution after relaxation in the potential energy function.

This procedure was run iteratively as more terms were added to the potential energy function. The final 4-mer pair reference energy term was introduced to reproduce the same consecutive pair frequencies as observed in the PDB and parameterized in a similar way:

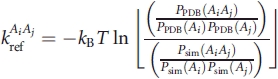
(7)

The α-carbon-α-carbon pseudobond term was similarly approximated as a harmonic potential and parameterized in the same way as the bond angle terms. Two sets of α-carbon-α-carbon pseudobond terms were defined—one for *trans* peptide bonds and one for *cis* peptide bonds.

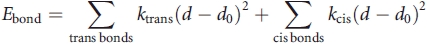
(8)

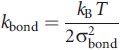
(9)

The soft α-carbon-α-carbon steric repulsive term was of the form

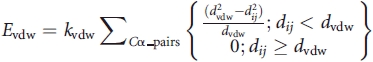
(10)where *k*_vdw_ was set to an arbitrarily high value (10 *k*_B_*T*) and *d*_vdw_ was set to 4 Å.

Each structure in the training set was randomized (setting dihedrals and bond angles to random values) and relaxed in the potential energy functions defined above by running Monte Carlo simulations for 1 million steps producing a set of noncompact random walk chains. The functions

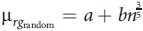
(11)and

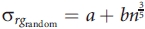
(12)where *n* is the number of residues]were found to very roughly fit the resulting distribution in this size range. Similarly for the compact domains in the training set the functions

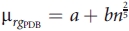
(13)and

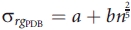
(14)were found to roughly fit the observed distribution. Using these fitted parameters and using a Gaussian probability density function as an approximation, the final energy function takes the form

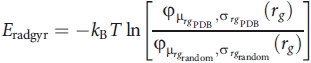
(15)which can be simplified to

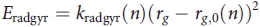
(16)

Given the large approximations involved, small changes to these parameters had to be made by hand in order to get folds of a compact globular nature.

A set of knowledge-based directional and distance-dependent hydrogen bonding terms were also defined. This utilized pseudoatoms N′ and O′ as defined by Levitt^37^ [Fig. 1(b)]. Each 3-mer defined a set of N′ and O′ atoms. Each N′_*i*_ was defined as being midway between Cα_*i*_ and Cα_*i*+1_. O′_*i*_ was defined as 1 Å from N′_*i*_ in the direction perpendicular to the plane defined by Cα_*i*_, Cα_*i*+1_, and Cα_*i*+2_. A pair of N′ and O′ atoms was defined to be hydrogen bonded if they were less than 4.5 Å and more than 3.0 Å apart. Four classes of hydrogen bond types were defined—(i) the hydrogen bonding “letters” were both of the helical class and with a sequence separation of 3 (not 4 due to the numbering scheme), (ii) the hydrogen bonding letters were both of the strand class and with a sequence separation of between 4 and 5, (iii) the hydrogen bonding letters were both of the strand class the sequence separation was more than 5, and (iv) all other cases with a sequence separation of more than 3.

The training set of PDB structures were randomized (setting dihedrals and bond angles to random values) then relaxed in the potentials defined above to provide background distributions of distances and angles. As the potential energy function now includes the term *E*_radgyr_, the resulting structures resemble random walks of globular domain compactness. The distance dependent term for each hydrogen bonding class was defined as:

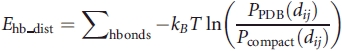
(17)

As the angular distributions were clearly distance-dependent (especially in the short range hydrogen bonding classes), the angular frequency counts of the background distribution, *P*_compact_, were weighted by the Boltzmann factor:

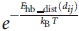
(18)to correct for the effects of distance on the angular distributions resulting in the modified distribution *P*′_compact_. As a simplifying assumption, we considered the angular distributions to be independent of each other. The angle terms were defined as:

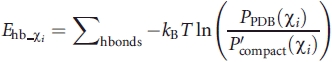
(19)

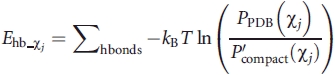
(20)

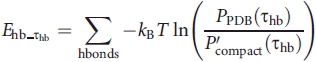
(21)

In addition to the distance and angular terms, there was also a reference energy related to how probable a hydrogen bond was to form in the training set compared to the random compact globular background set. This term was also calculated separately for each of the four classes and defined as:

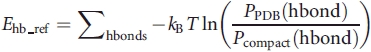
(22)

Finally, because there were no explicit terms to account for secondary structure element packing and to restrict refinement to explore only the region around the desired tertiary fold, three extra terms were added to the potential energy function.

Line segments corresponding to each defined secondary structure element were calculated by finding the least squares fit to the α-carbon atoms in the element. To maintain good secondary structure packing, these elements were restrained to the positions in the ideal forms by restraining the closest distances to the ideal line segments and the angles to the ideal line segments:

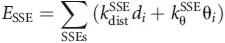
(23)

To restrain the residues predefined as part of a helix or strand to compatible conformations, a term to restrain α-carbon pseudotorsion and bond angles to ideal helical or strand values was defined:

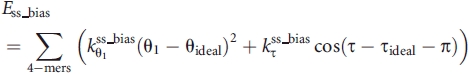
(24)where the ideal angles were determined by finding the medians from secondary structure elements in the training set and the force constants set by a trial and error process.

To keep the strands of the β-sheets in close proximity during the initial stages of refinement and to prevent the structure from “blowing up” a further restraint was added:

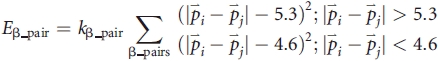
(25)

### Main-Chain Potential Energy Function

The main-chain potential energy function was designed solely to ensure good local backbone stereochemistry and is therefore a very simple hybrid of terms derived from the OPLS-UA force field,^35^ the Rosetta force field, and the α-carbon radius of gyration term described above.

All bonded parameters (bond, torsion, improper torsion, 1-4 Lennard-Jones, and 1-4 electrostatic) were taken directly from the OPLS-UA force field. In addition to these bonded terms, the Lennard-Jones and electrostatic terms were also evaluated for 1-5 and 1-6 atom pairs. For atom pairs separated by more than five bonds, a simple soft steric repulsive term was evaluated:

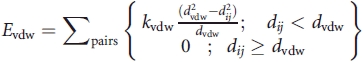
(26)where the diameters, *d*_vdw_, for each atom pair type were taken from the Lennard-Jones parameters in the OPLS-UA force field. It was found that some atom pair types clashed frequently in high-resolution PDB structures. These diameters were reduced to ameliorate this problem.

The hydrogen-bonding potential was a direct reimplementation of the Rosetta hydrogen bonding potential with linear interpolation between the distance and angular bins to allow gradient calculations for minimization.^33^,^34^

### The High-Resolution Training Set

The training set of high resolution experimentally determined structures was taken from SCOP 40 v1.73 using only X-ray structures with SPACI scores of more than 0.4, with complete resolved backbones and of chain lengths between 50 and 200 residues.^38^ This resulted in a training set of 2285 structures.

## RESULTS

The methods described earlier were used to produce a set of 9000 main-chain decoy backbones (referred to as “decoys”) of 72 residues long and setting the predefined secondary structure from the Atx1 metallochaperone (PDB code: 1CC8): 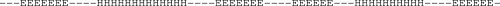


The secondary structure and length were chosen to be long enough to produce a number of nontrivially different topologies but short enough to make sequence design of a large number of backbones to be computationally tractable. This resulted in 26 unique topologies (Table III). As controls, 17 real protein backbone domains (referred to as “real”) of the same residue length from the PDB (Table I) and 2000 compact random walk structures (referred to as “random”) were put through the same Rosetta design/relaxation protocol. Each of the 17 real backbone scaffolds was redesigned 100 times generating a total of 1700 sequences and structures. The compact random walk structures were generated by relaxing random walks in the α-carbon potential energy function with all hydrogen bonding terms turned off to prevent secondary structure formation then adding main-chain atoms with the usual protocol. This procedure produced compact random coil structures with radii of gyration similar to single compact domains. The real backbone scaffold set was produced to determine the redesigned energy distributions of backbones that were known to be “designable,” whereas the compact random set would give the energy distributions of structures with arbitrarily bad tertiary folds of globular domain compactness and good dihedral angles and were therefore assumed to be “undesignable.”

**Table I:** Real Protein Controls

PDB code	Residues	Chain	Radius of gyration/Å	Experimental method	Resolution/Å	Rosetta energy after relaxation
2jdi	10–81	D	10.39	XTAL	1.90	−150.74
2bwf	2–73	A	10.49	XTAL	1.15	−154.59
2as0	1–72	A	10.50	XTAL	1.80	−164.50
1osd	1–72	A	10.70	XTAL	2.00	−152.30
1ubq	1–72	A	10.71	XTAL	1.80	−166.36
1wm3	17–88	A	10.87	XTAL	1.20	−154.52
1hyp	6–77	A	10.88	XTAL	1.80	−115.45
1cc8	2–73	A	10.91	XTAL	1.02	−151.57
4ait	3–74	A	10.91	NMR	n/a	−122.73
1o8b	127–198	A	10.92	XTAL	1.25	−146.61
1lea	1–72	A	10.96	NMR	n/a	−153.41
1zyb	149–220	A	11.12	XTAL	2.00	−156.17
1v97	94–165	A	11.17	XTAL	1.94	−109.02
1vcc	1–72	A	11.30	XTAL	1.60	−161.37
1iyu	1–72	A	11.31	NMR	n/a	−139.04
1i27	445–516	A	11.81	XTAL	1.02	−150.68
1dzf	144–215	A	12.05	XTAL	1.90	−146.29

Rosetta energies of the wild-type sequences are given after idealization and one round of Rosetta relaxation.

**Figure 2:**
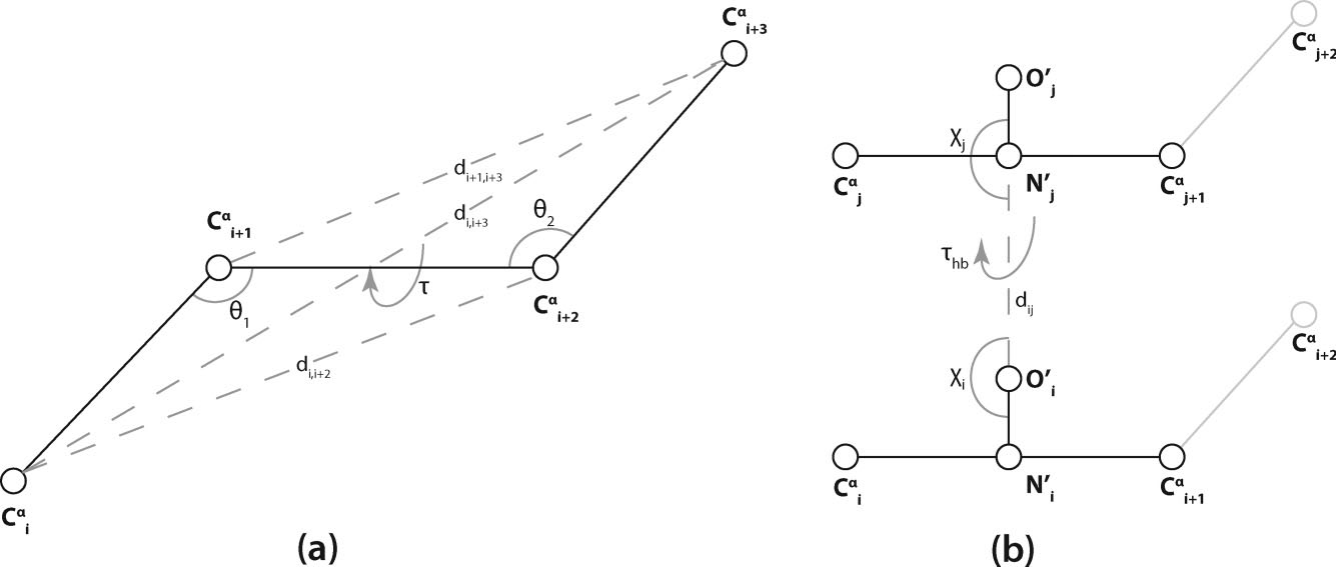
α-Carbon 4-mer and pseudohydrogen bond geometry. (**a**) 4-mer letter angles and distance bins. (**b**) Pseudohydrogen bonding where τ_hb_ is the dihedral angle defined by Cα_*i*_ − N′_*i*_ − N′_*j*_ − Cα_*j*_. O′ is defined to be 1 Å from N′ in the direction (**Cα**_*i*+1_ − **Cα**_*i*_) × (**Cα**_*i*+2_ − **Cα**_***i*+1**_).

**Figure 3:**
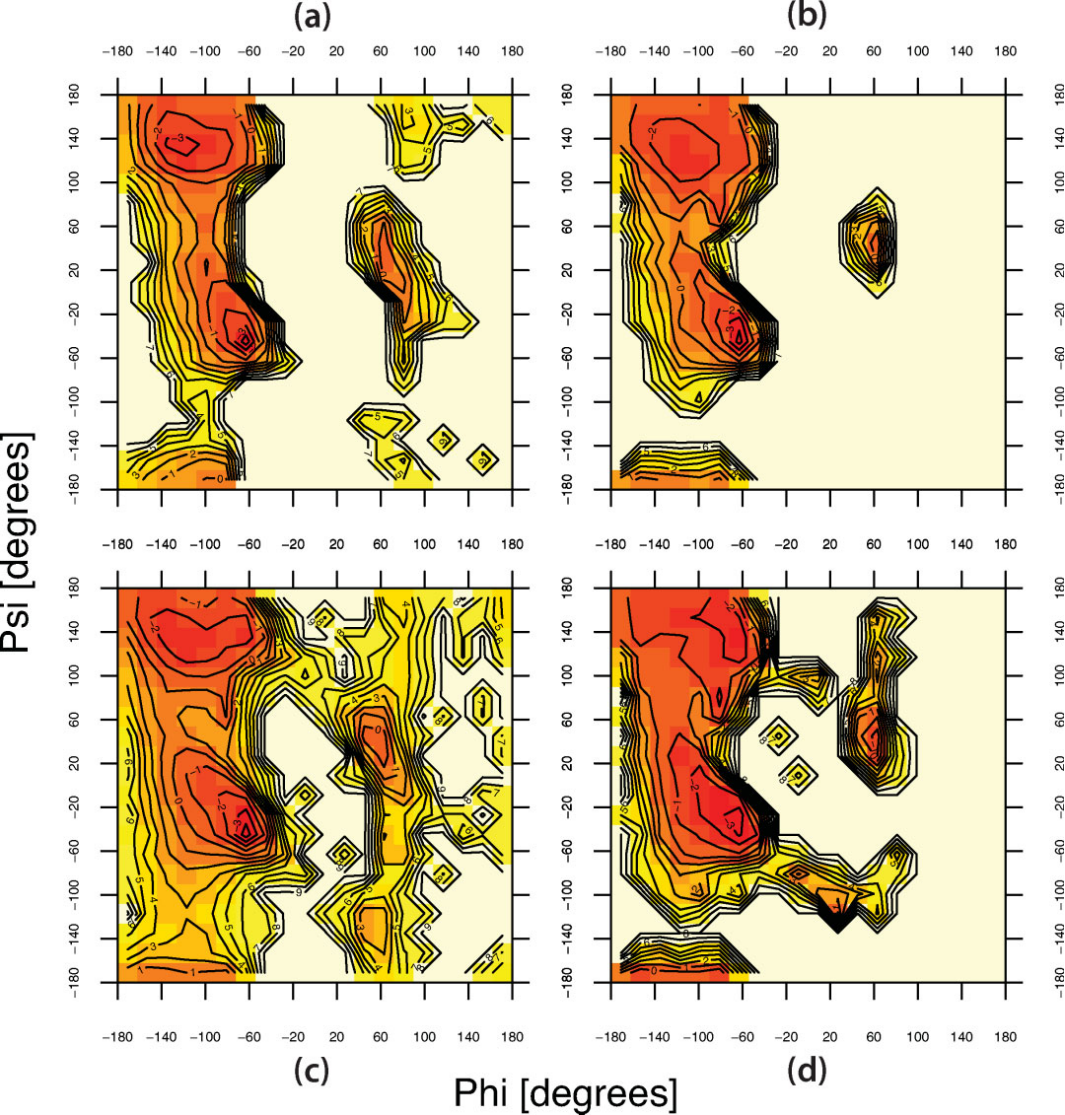
Log likelihood Ramachandran distributions of (**a**) the novel scaffold decoys (“decoys2”) before Rosetta design/relax, (**b**) the novel scaffold decoys (“decoys2”) after Rosetta design/relax, (**c**) the high-resolution PDB training set, and (**d**) the compact random coil controls (“random”) after Rosetta design/relax. For the log likelihood distributions for “decoys” see Supporting Information Figure S1.

A second set of decoys (referred to as “decoys2”) and real (referred to as “real2”) redesigned backbones were produced using a slightly modified protocol. The new “decoys2” set differed from the original “decoys” set by applying two sets of filters before the more computationally intensive Rosetta design/relax cycles. The first filter was applied at the initial α-carbon model stage. Using the N′ and O′ atoms as defined in Figure 2(b) and crudely defining a pseudohydrogen bond where the distance between these atoms is less than 5 Å, the proportion of predefined sheet and helix residues involved in hydrogen bonding was counted. If either of these counts was below 18%, the model was filtered out. A similar filter was put in place after the main-chain reconstruction step. In this case, if the percentage of either helical or strand secondary structure fell below 25%, the sum of the two fell below 60% or if more than one of the residues was in the disallowed region [defined as the region in Fig. 3(c) where the negative log likelihood is above a threshold of 4] of the Ramachandran plot, then the model was filtered out. The Rosetta design/relax stage was also modified by running a reduced number of cycles (10 instead of the original twenty cycles) and by constraining the top 10% of buried residues to be hydrophobic and bottom 10% to be hydrophilic with the extra condition that the residue is in either an α-helix or a β-strand where burial was defined as the number of Cβ atoms in a 9 Å sphere around each Cβ atom. Constraining the most solvent exposed and least solvent exposed residues was found to significantly decrease the solvation energy term. The “decoys2” set consisted of 1000 structures, whereas the “real2” set consisted of 1700 structures (the same as the original “real” set).

**Figure 4:**
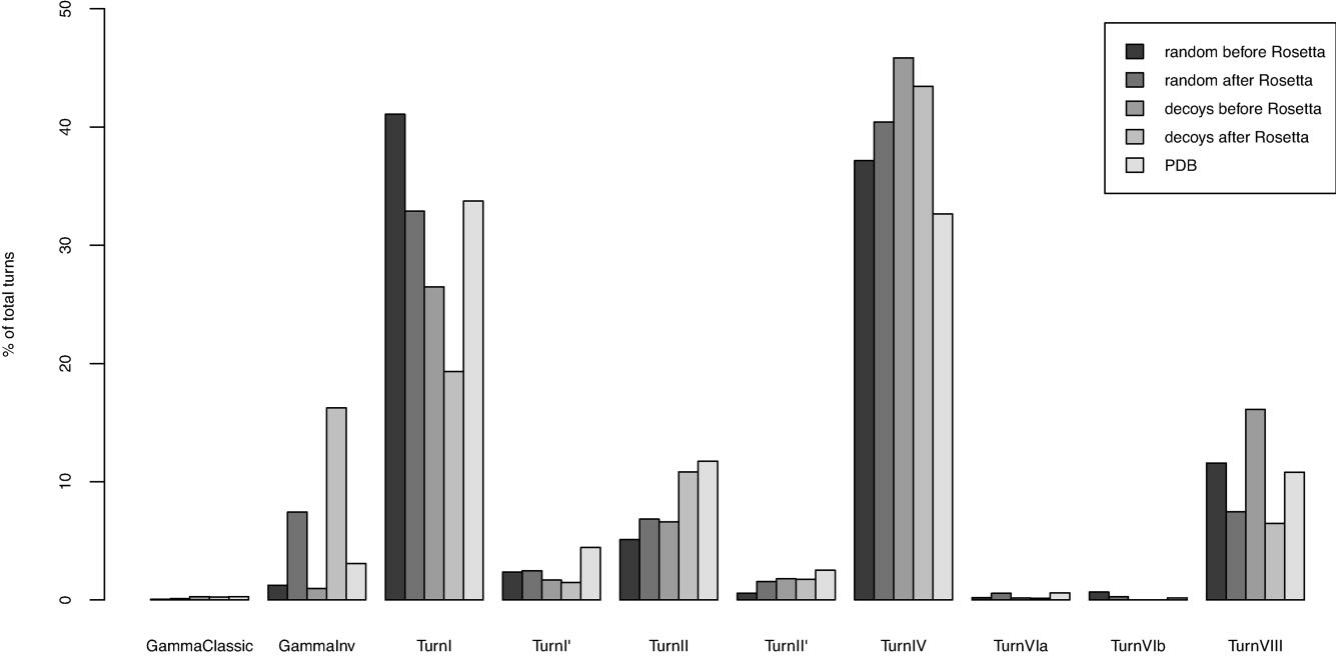
Relative β- and γ-turn frequencies in the novel scaffold decoys, compact random controls, and the high resolution PDB training set as assigned by STRIDE.^39^

Local main-chain conformations for all the resulting structures were found to be similar to real proteins (Figs. 3 and 4 and Supporting Information Fig. S1). The distribution of dihedral angles showed a clear preference for the most favorable regions of the Ramachandran plot after the initial main-chain construction protocol [Fig. 3(a)] with most outliers removed after Rosetta design/relaxation [Fig. 3(b)]. The distribution of turns (as determined by STRIDE^39^) was also found to be similar to the real PDB [Fig. 4(a)]. This is likely a direct result of the use of the structural alphabet in α-carbon potential energy function and the parameterization procedure.

In the absence of any other metric, it was decided to use the Rosetta energy function to evaluate the “designability” of the novel scaffolds. Real protein main chains were assumed to have evolved, such that good side-chain packing could occur in the core of the protein and the number of buried unsatisfied hydrogen bonds minimized. In contrast, it was assumed that compact random coils do not have these properties and therefore have minimal “designability.”

**Figure 5:**
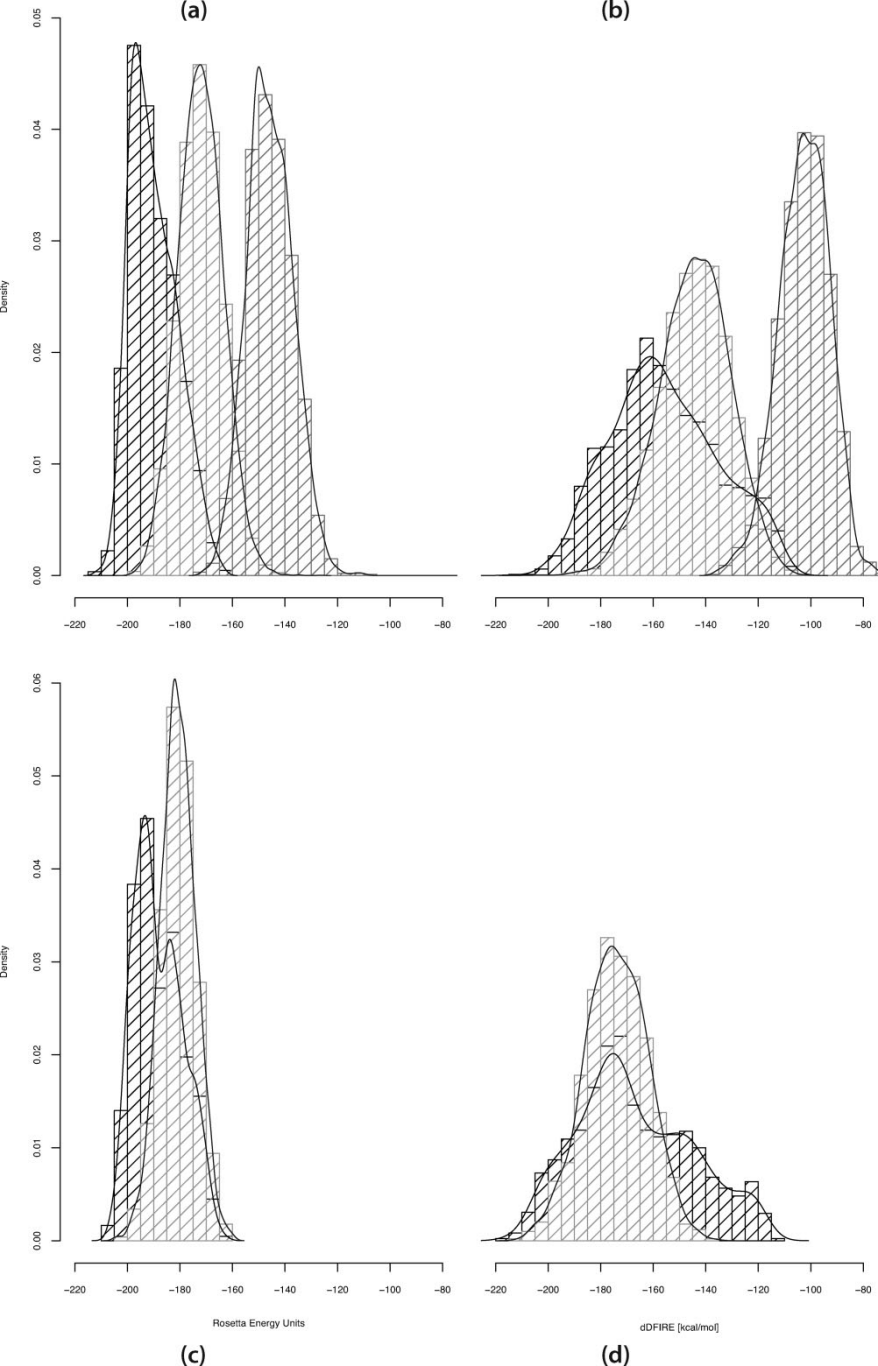
Overall (**a**, **c**) Rosetta and (**b**, **d**) dDFIRE energy histograms for the real protein controls (black), novel decoy scaffolds (light gray), and the compact random coil controls (dark gray). (**a**, **b**) Show the energies for the “real,” “decoys,” and “random” original design protocol sets, whereas (**c**, **d**) show the energies for the “real2” and “decoys2” design protocol sets.

Overall, the Rosetta energies of the “decoys” scaffolds lie in between the real main chains and the random coils [Fig. 5(a)] but with greater overlap with the “real” main-chain designs than with the “random” coil designs. The dDFIRE energy distribution was also calculated as an independent potential energy function [Fig. 5(b)]. This confirmed a high degree of overlap between the decoys and the real backbone design energy distributions. The filtered “decoys2” energies show a much greater overlap with the “real2” backbone design energies [Fig. 5(c,d)] with the high-energy tails eliminated and the peaks shifted substantially to the left. The dDFIRE histogram appears to show that the “real2” set has more of a high-energy tail than the “decoys2” set.

Comparing the distributions of the Rosetta energy function components of real main chains and the random coils, it was seen that the terms related to side-chain packing (i.e. fa_atr (Lennard-Jones attractive term)) and hydrogen bonding (i.e., the hbond_sr_bb, hbond_lr_bb, hbond_bb_sr, and hbond_sc terms) were indeed significantly different as expected (Supporting Information Table S1). Interestingly, although backbone-backbone hydrogen bonding was worse, backbone-side-chain and side-chain-side-chain hydrogen bonding was better. This could be due to the increased number of buried unsatisfied backbone donors and acceptors in the random coil structures.

Breaking down the contributions of the different terms in the potential energy function, it was seen that the difference between the “real” backbones and the “decoys” is mainly due to higher long-range backbone-backbone hydrogen bonding energy and higher solvation energy (Supporting Information Table S1). This suggests that on an average, the “decoys” scaffolds have fewer and/or worse hydrogen bonding in the β-sheets than in real main-chain structures; however, it should be noted that the novel scaffolds include topologies that are not necessarily favorable with the predefined secondary structure. Indeed, a large degree of variation in mean long-range backbone-backbone hydrogen bonding energies was observed when divided by topology (Table III). The higher solvation energy suggests that some of the decoys do not have as well defined hydrophobic cores. To a lesser extent, the terms “fa_atr” and “rama” were also on average worse perhaps reflecting slightly less favorable side-chain packing and backbone torsion angles. The second set of filtered decoys, “decoys2,” was found to have solved the problem of higher long-range hydrogen bonding energies. Overall, the difference in mean total energies between the “decoys2” and “real2” sets was found to have reduced to −7.49. This difference was not attributable to any one dominant term but is the result of many small differences in the individual terms (Table II). This suggests that the “decoys2” set are of a high quality across a broad range of measures.

**Table II:** Rosetta Energy Constituents of “Real2” and “Decoys2”

Rosetta energy term	Physical meaning	μ_real2_	μ_decoys2_	Δ_real2-decoys2_	*p*-value
fa_atr	Lennard-Jones attractive	−285.29	−283.05	−2.24	2.96E-04
fa_rep	Lennard-Jones repulsive	27.01	25.76	1.24	<2.20E-16
fa_sol	solvation energy	126.78	124.80	1.98	1.28E-06
fa_intra_rep	Intraresidue LJ repulsive	0.70	0.75	−0.04	<2.20E-16
pro_close	Proline ring closure	0.05	0.02	0.02	<2.20E-16
fa_pair	Statistical pair energy	−10.27	−9.86	−0.41	2.88E-03
hbond_sr_bb	Backbone-backbone hbonds close in primary sequence	−15.27	−15.26	−0.01	0.94
hbond_lr_bb	Backbone-backbone hbonds distant in primary sequence	−27.09	−28.05	0.96	6.15E-03
hbond_bb_sc	Side chain-backbone hydrogen bond energy	−7.88	−5.49	−2.39	<2.20E-16
hbond_sc	Side chain-side chain hydrogen bond energy	−7.52	−7.60	0.08	0.54
Rama	Ramachandran energy	−7.50	−6.09	−1.41	<2.20E-16
Omega	Omega dihedral energy	4.80	5.79	−0.99	<2.20E-16
fa_dun	Internal energy of sidechain rotamers	38.03	39.37	−1.33	3.97E-12
p_aa_pp	Amino acid Phi-Psi statistical energy	−10.24	−9.11	−1.13	<2.20E-16
ref	Amino acid reference energy	−14.59	−12.77	−1.81	1.65E-14
Total	Sum of all terms	−188.29	−180.80	−7.49	<2.20E-16

For the energy constituents of “real,” “decoys,” and “random,” see Supporting Information Table S1.

Mean amino acid compositions for the “real,” “real2” (1700 sequences, 124,100 residues), “random” (2,000 sequences, 146,000 residues), “decoys” (9000 sequences, 648,000 residues), and “decoys2” (1000 sequences, 72,000 residues) structures were compared with the mean composition for ASTRAL SCOP40 (9536 sequences, 1,716,774 residues) using the nonparametric Spearman correlation coefficient with cysteine excluded (because RosettaDesign never produced this residue). Residue compositions for the “real” structures were significantly correlated with the ASTRAL compositions (ρ = 0.70, *t*= 4.08, *p* < 0.001, 17 d.f.) as were the compositions for the “decoys” (ρ = 0.55, *t* = 2.72, *p* < 0.01, 17 d.f.), the compositions for “real2” (ρ = 0.66, *t* = 3.59, *p* < 0.01, 17 d.f.), and the compositions for “decoys2” (ρ = 0.53, *t* = 2.61, *p* < 0.05, 17 d.f.). Sequences for the random designs were not significantly correlated (ρ = 0.35, *t* = 1.54, *p* > 0.05, 17 d.f.). The *t* values were calculated using the formula:

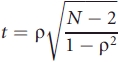
(27)

All three groups of the original protocol set (“real,” “decoys,” and “random”) were observed to be depleted in Met, Phe, Pro, and Trp (Supporting Information Table S2, Fig. S2). The compact “random” structures were highly enriched in small and polar amino acids (Asp, Gly, and Ser) and depleted in certain hydrophobic residues (Val and Ile), suggesting a lack of good core packing and excessive solvent exposure. The sequences of the novel “decoys” showed some of these features but were overall most similar to the sequences designed for real structures.

We also examined the compositions for the modified protocol set (“real2” and “decoys2”). In these cases, the compositions must be assumed to be less informative of the overall quality of the structures because the compositional identity was constrained for 20% of the residues per iteration. Overall, we found that Pro, Ser, Thr, and Val were strongly disfavored, with both the decoys and the real structures substantially depleted in these residues and the decoys especially depleted in Pro (Supporting Information Table S3, Fig. S3). A bias toward Trp, Tyr, Lys, and Arg was also apparent for both sets with little difference between the two.

A few residues differed in composition between the “real2” and “decoys2” sets. Ala and Asp were in excess in “decoys2” but not in the “real2” set. Phe was in excess in both sets but to a much greater extent in “decoys2.” Gly was somewhat depleted in both but much more strongly in the decoys. Leu was depleted in the decoys but in excess in the designs based on real structures. Most of the differences are minor, the most significant being Pro, Gly, and Phe. The differences in Gly content may be explained by the filtering of disallowed dihedral angles. However, the increase in Phe content is more difficult to explain.

The compositions of the “real2” and “decoys2” were further broken down by secondary structure type (Supporting Information Figs. S4 and S5). The β-strand compositions were roughly similar between the two sets with the “decoys2” enriched in Phe, Ile, and Ala. More substantial differences were observed in the α-helical compositions with both sets greatly enriched in Glu, Lys, Arg, and Trp. The “decoys2” set was found to be enriched in Ala but depleted in Ile, Leu, and Val. This could suggest that the novel scaffold helices have a tendency to be too tightly packed to allow room for the larger hydrophobic residues.

BLAST searching of all designed sequences against the nonredundant protein sequence database (nr, July 2009) filtered for low-complexity regions detected no significant similarities between the nonreal backbone designed sequences and real protein sequences, but some of the real backbone redesigns did have significant similarities to protein sequences from corresponding folds.

**Figure 6:**
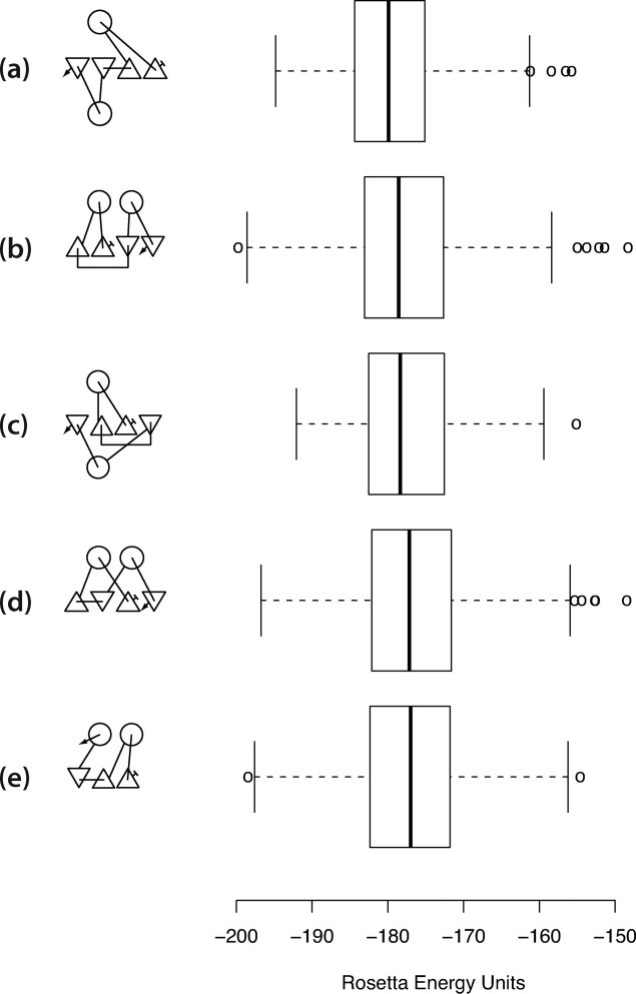
Top 5 median Rosetta energy topologies where (**a**) corresponds to topology index 9 from Table III, (**b**) topology index 2, (**c**) topology index 8, (**d**) topology index 1, and (**e**) topology index 15.

The “decoys” set was found to have a range of between −179.89 and −160.77 median Rosetta design energies when split by topology was observed (Table III), and it is striking that the topologies with the lowest Rosetta energies (top half of Table III) also tend to have the greatest number of topology matches in SCOP 40. As one would expect the Ferredoxin-like fold of the metallochaperone that provided the initial secondary structure was found among the top ranking topologies, and surprisingly, topologies with the two helices separated on opposite sides of the β-sheet do not seem to have been penalized despite having less opportunity to make a compact hydrophobic core (Fig. 6).

**Table III:** Topology Hits and Top SAP Matches to SCOP 40 with the “Decoys” Set

					Top matching SCOP domain by SAP structural alignment			
Topology index	Topology string	Median Rosetta energy	Mean hbond_lr_bb Rosetta energy term	No. of topology hits in SCOP 40	SCOP ID	RMSD/Å	No. of aligned residues/72 possible	Rosetta energy of best aligned decoy
9	+B+0.-A+0.+B-1.-B-2.+C+0.-B-3.	−179.89	−26.63	96	d1u0ka1	3.26	65	−182.87
2	+B+0.-A+0.+B-1.-B+1.+A+1.-B+2.	−178.58	−22.63	45	d1mwwa_	2.49	68	−182.59
8	+B+0.-A+0.+B-1.-B+1.+C+0.-B-2.	−178.36	−24.67	0	n/a	n/a	n/a	n/a
1	+B+0.-A+0.+B-2.-B-1.+A+1.-B+1.	−177.17	−24.13	302	d1ukua_	1.79	65	−189.45
15	+B+0.-A+0.+B-1.-B-2.+A-1.	−176.98	−18.72	133	d1q0pa_	3.19	68	−181.52
4	+B+0.-A+0.+B-2.-B-1.+A+1.	−176.73	−17.33	464	d1nm2a2	2.06	61	−185.28
5	+B+0.-A+0.+B-1.-B+1.+A+1.	−174.14	−14.92	118	d1us5a_	2.82	69	−185.23
16	+B+0.-A+0.+B-1.-B-2.-A-1.	−173.18	−15.14	87	d1d6aa_	2.69	58	−186.66
11	+B+0.+A+0.-B-1.+B-2.-A-1.	−170.51	−13.64	46	d1hr6b2_	3.01	66	−172.04
24	+B+0.-A+0.-B+1.+B-1.+C+0.-B+2.	−168.82	−18.16	0	n/a	n/a	n/a	n/a
21	+B+0.+A+0.-B-2.+B-1.-A+1.	−168.60	−13.43	0	n/a	n/a	n/a	n/a
36	+B+0.+A+0.-B-2.+B-1.-A+1.	−167.63	−10.33	0	n/a	n/a	n/a	n/a
27	+B+0.-A+0.-B-1.+B-2.+C+0.-B+1.	−167.54	−19.43	0	n/a	n/a	n/a	n/a
35	+B+0.+A+0.-B-1.+B-2.+C+0.	−167.41	−13.32	66	d1cjwa_	5.36	64	−168.08
17	+B+0.-A+0.-B+1.+B+2.+C+0.-B-1.	−167.37	−19.91	0	n/a	n/a	n/a	n/a
12	+B+0.-A+0.-B-1.+B-2.-A-1.	−167.36	−14.65	42	d1kfsa1	3.43	57	−160.37
20	+B+0.-A+0.-B+1.+B-1.+C+0.-B-2.	−166.95	−16.65	0	n/a	n/a	n/a	n/a
26	+B+0.-A+0.-B+1.+B+2.+A+1.	−166.62	−13.79	0	n/a	n/a	n/a	n/a
13	+B+0.+A+0.-B+1.+B-1.-A-1.	−166.36	−8.85	0	n/a	n/a	n/a	n/a
23	+B+0.-A+0.-B-2.+B-1.+C+0.-B+1.	−166.35	−19.25	5	d1cjxa1	3.69	61	−176.03
18	+B+0.-A+0.-B+2.+B+1.+C+0.-B-1.	−165.87	−13.64	0	n/a	n/a	n/a	n/a
31	+B+0.-A+0.-B+1.+B-1.+C+0.	−165.33	−13.70	0	n/a	n/a	n/a	n/a
25	+B+0.-A+0.-B-1.+B-2.+A-1.	−165.12	−13.72	0	n/a	n/a	n/a	n/a
30	+B+0.-A+0.-B+1.+B-1.+A-1.	−163.43	−10.55	0	n/a	n/a	n/a	n/a
34	+B+0.-A+0.-B+2.+B+1.+A-1.-B-1.	−163.12	−18.54	0	n/a	n/a	n/a	n/a
28	+B+0.-B+1.+B+2.+A-1.-B-1.	−160.77	−23.00	0	n/a	n/a	n/a	n/a

The “topology string” encodes a given backbone topology as a unique string. Each structure can be matched to an “ideal form” and the path the secondary structure elements (SSEs) take over this “ideal form” then describes the topology. For a three layer, α-β-α protein, a simple coordinate system, was used where each SSE was assigned to a layer (“A,” “B,” or “C”) with a relative orientation (“+” or “−”) and a relative position within the layer (“−1,” “+0,” “+1,” “+2,” etc.). By convention, the first SSE to enter a layer was assigned the relative position “+0” with all other SSE in that layer numbered relative to that. The first strand in a sheet was given a positive orientation, so that the first strand in the string was always “+B+0.” The first helix was assigned to layer “A.” The full topology matching procedure is described by Taylor *et al*.^20^

To further probe the core side-chain packing, the structures were scored with RosettaHoles, a method to assess and visualize protein core packing by generating groups of cavity-filling balls.^40^ The overall RosettaHoles score is the sum of the predicted RMSD and 3 × the predicted probability of the model not being from a high-resolution crystal structure. The “real” (4.56 ± 0.54) and “real2” (4.56 ± 0.64) backbone designs had better overall mean scores than both the “decoys” (4.74 ± 0.53), “decoys2” (4.8 ± 0.39), and the “random” controls (4.88 ± 0.54). These results are not statistically significant, but the RosettaHoles score may prove to be useful to pick candidates for experimental study.

**Figure 7:**
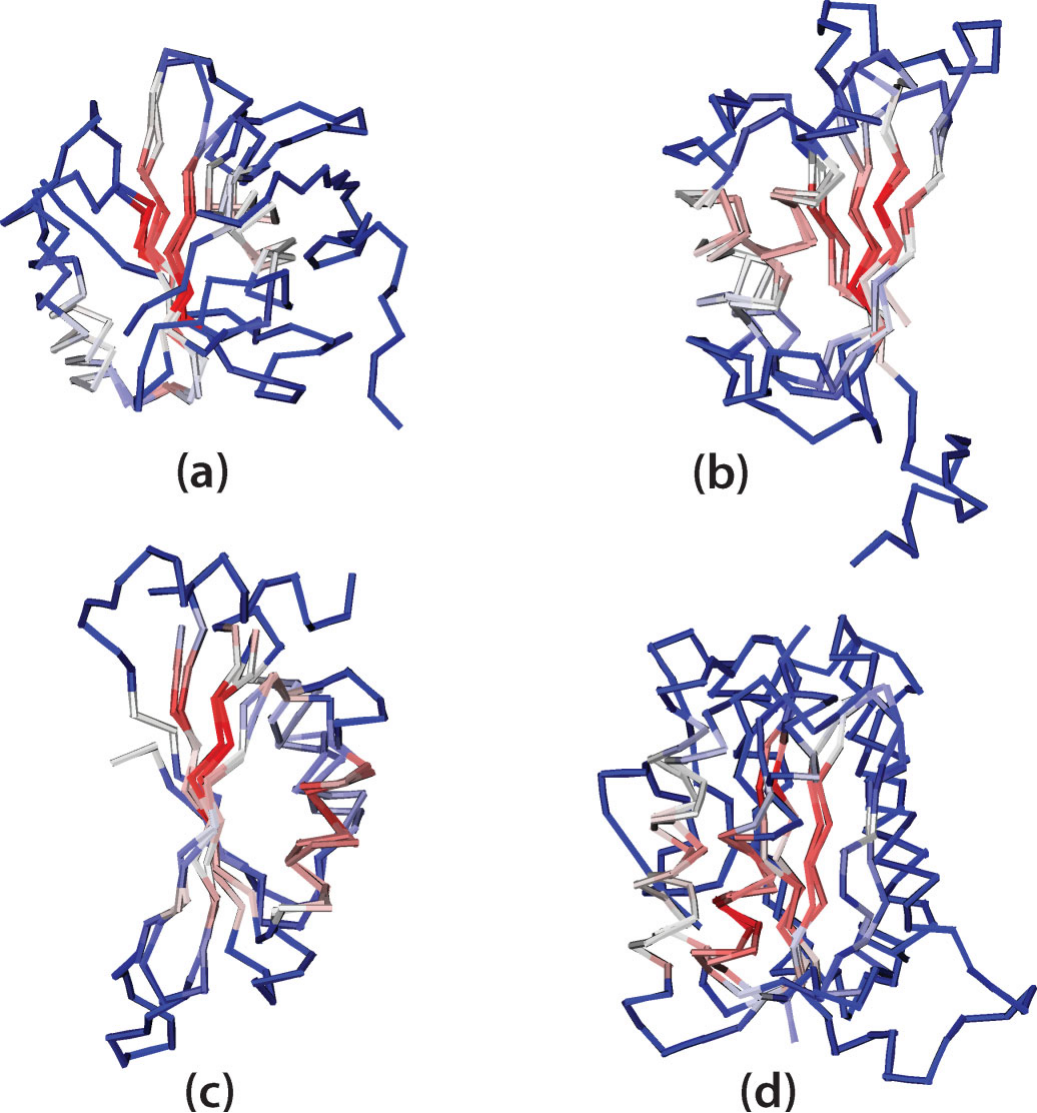
Top four SAP alignments from Table III where (**a**) corresponds to topology index 9, (**b**) topology index 2, (**c**) topology index 1, and (**d**) topology index 15. This figure was produced using VMD.^42^

As a final test of the method's overall ability to reproduce protein-like tertiary folds, structural alignments with known protein structures were carried out. All structures from the “decoys” set were structurally aligned using SAP^41^ with all matching topology hits found in SCOP 40. For each unique topology, the best aligned SCOP 40 domains were recorded (Table III; Fig. 7). Given the vast size of structure space, it is surprising that from just 9000 decoy structures, a number of very close structural alignments were found across a number of different folds. The best result is an alignment of 1.79 Å RMSD covering 90% of the residues for the Ferredoxin-like fold, and there are also a number of other sub-3 Å alignments. It can be seen by visual inspection of the structural super-positions that the β-sheets of the decoys align well with β-sheets from the experimental structures (Fig. 7).

## DISCUSSION

The coarse-grained hierarchical backbone construction method we have presented has been demonstrated to produce realistic backbone models in terms of the Ramachandran plot (Fig. 3), relative turn frequencies (Fig. 4), overall tertiary structure (Fig. 7), and amino acid composition after RosettaDesign.

We have assessed the “designability” of the backbones by comparing the potential energy distributions of the decoy structures with real protein backbone structures after 20 rounds of design and relaxation using Rosetta (Figs. 5 and 6; Tables II and III). Although this is not strictly theoretically justified, we believe this is a useful heuristic within the paradigm of positive design methods where sequence and structure are simultaneously optimized in a potential energy function. A significant proportion of the designed decoys were within the range of the redesigned real backbones. Within this set, it is hoped that at least a proportion are experimentally foldable. The difference between the decoy and real backbone energy distributions was found to be mainly due to worse long-range backbone-backbone hydrogen bonding energies on average and was found to be easily solvable by filtering out bad models at earlier stages. The long-range hydrogen bonding energies were found to be highly dependent on topology and most of the top ranking topologies showed good β-sheet formation (Table III).

A method to sample rapidly a wide variety of designable backbone conformations could help provide solutions to the large number of remaining problems in computational protein design. In the first instance, this method could generate a large library of backbone scaffolds that could then be scanned for potential catalytic sites using existing methods such as RosettaMatch.^43^ Although previous work has shown it is possible to design *de novo* protein dimer interfaces,^44^ large-scale backbone sampling may also be necessary for the design of novel protein-protein and protein-DNA interfaces with naturally occurring targets.^45^,^46^

In the near-term progress in computational enzyme design is likely to come from the local remodeling of backbone regions near the active site. However, we propose that as the target functions of designed proteins become more ambitious, the less likely it is that existing scaffolds are able to satisfy all the backbone constraints without remodeling large parts of the scaffold. A method of systematically generating a large number of *de novo* backbone scaffolds may eventually prove to be an efficient way of solving this problem. If one could annotate the dynamical propensities (e.g. by an analysis of normal modes or some other method) of a given backbone loop conformation in a training set of known proteins, it may be possible to search for possible backbone scaffolds that are more likely to be compatible with particular desired conformational changes. The method could find applications in synthetic biology (e.g. it could be used to design linkers between functional subunits) and in the design of novel materials.

The question of whether certain protein topologies that have not been experimentally observed do not exist for some physical reason rather than an evolutionary reason could be addressed using computational protein design.^20^ If Nature has only explored a limited region of fold space due to limited need or not having had enough time, then it should be possible to design and experimentally fold these novel structures given an appropriate method in addition to vastly expanding the range of possible scaffolds for enzyme design.
