# Supplementary material for: De novo backbone scaffolds for protein design
Source: Proteins. 2009 Nov 5;78(5):1311–25. doi: 10.1002/prot.22651 (PMC2841848; doi:10.1002/prot.22651)
Supplement: Supplementary file 1 [file prot0078-1311-SD1.pdf]

Supporting Information for "*De novo* backbone scaffolds for protein design"

Rosetta Energy term	μ_{Real}	μ_{Decoys}	μ_{Random}	$\Delta_{Real-Decoys}$	p-value Real-Decoys	$\Delta_{Real-Random}$	p-value Real-Random	$\Delta_{Decoys-Random}$	p-value Decoys-Random
**fa_atr**	-285.63	-281.83	-256.82	-3.81	<2.20E-16	-28.81	<2.20E-16	-25.01	<2.20E-16
**fa_rep**	25.70	25.43	28.28	0.27	9.17E-04	-2.58	<2.20E-16	-2.85	<2.20E-16
**fa_sol**	134.36	140.37	140.01	-6.01	<2.20E-16	-5.65	<2.20E-16	0.36	2.60E-01
**fa_intra_rep**	0.65	0.64	0.55	0.00	1.42E-01	0.10	<2.20E-16	0.10	<2.20E-16
**pro_close**	0.05	0.03	0.05	0.02	2.86E-16	-0.01	4.00E-02	-0.03	<2.20E-16
**fa_pair**	-11.66	-13.73	-13.28	2.07	<2.20E-16	1.62	<2.20E-16	-0.45	5.33E-06
**hbond_sr_bb**	-15.49	-16.70	-9.04	1.21	6.47E-11	-6.44	<2.20E-16	-7.65	<2.20E-16
**hbond_lr_bb**	-27.57	-16.45	-7.12	-11.12	<2.20E-16	-20.45	<2.20E-16	-9.33	<2.20E-16
**hbond_bb_sc**	-8.75	-9.97	-20.00	1.22	<2.20E-16	11.25	<2.20E-16	10.03	<2.20E-16
**hbond_sc**	-8.81	-10.66	-10.62	1.85	<2.20E-16	1.81	<2.20E-16	-0.04	7.02E-01
**rama**	-7.36	-4.69	-3.48	-2.67	<2.20E-16	-3.88	<2.20E-16	-1.21	<2.20E-16
**omega**	4.62	5.09	4.59	-0.47	<2.20E-16	0.04	2.69E-01	0.51	<2.20E-16
**fa_dun**	35.61	37.53	32.97	-1.92	<2.20E-16	2.64	<2.20E-16	4.57	<2.20E-16
**p_aa_pp**	-10.67	-10.28	-12.58	-0.39	3.51E-14	1.91	<2.20E-16	2.30	<2.20E-16
**ref**	-15.04	-16.85	-19.07	1.82	<2.20E-16	4.03	<2.20E-16	2.21	<2.20E-16
**total**	-189.99	-172.07	-145.57	-17.92	<2.20E-16	-44.42	<2.20E-16	-26.51	<2.20E-16

Table S1: Rosetta energy constituents of "real", "decoys" and "random".

Residue	Astral	Decoy	Real	Random
A	8.18	8.19	8.58	5.36
D	5.88	10.28	7.50	11.88
E	7.05	8.38	7.84	4.83
F	4.07	5.61	4.90	3.81
G	7.43	6.51	7.36	12.38
H	2.35	6.02	4.43	6.85
I	5.79	3.79	5.55	1.46
K	6.04	8.86	8.67	6.34
L	9.42	7.12	9.78	5.83
M	2.21	0.87	1.12	0.53
N	4.26	3.48	3.00	5.61
P	4.68	0.45	0.93	0.93
Q	3.78	3.85	3.66	4.53
R	5.26	6.94	6.81	5.54
S	6.07	5.72	4.43	11.34
T	5.45	3.53	3.55	4.13
V	7.20	2.54	4.58	0.95
W	1.38	2.38	2.20	2.14
Y	3.49	5.48	5.11	5.55
**Total Res's**	**1668536**	**648000**	**122400**	**144000**

Table S2: Percentage residue compositions of the decoy set, random coil set and real design set for the original protocol. ASTRAL SCOP 40 compositions are provided as a comparison.

Residue	Astral	Decoy2	Real2
A	8.18	10.62	8.00
D	5.88	6.96	5.90
E	7.05	8.74	8.16
F	4.07	7.46	4.99
G	7.43	3.62	6.46
H	2.35	2.00	2.19
I	5.79	5.58	5.91
K	6.04	9.73	9.01
L	9.42	8.04	11.04
M	2.21	1.99	1.98
N	4.26	2.75	3.13
P	4.68	0.43	1.07
Q	3.78	3.45	3.81
R	5.26	6.96	7.34
S	6.07	3.68	3.25
T	5.45	2.61	2.93
V	7.20	4.85	5.27
W	1.38	3.69	3.04
Y	3.49	6.82	6.50
**Total Res's**	**1668536**	**72000**	**122400**

Table S3: Percentage residue composition of the two sets from the modified design protocol, with ASTRAL 1.73 SCOP40 as a comparison.

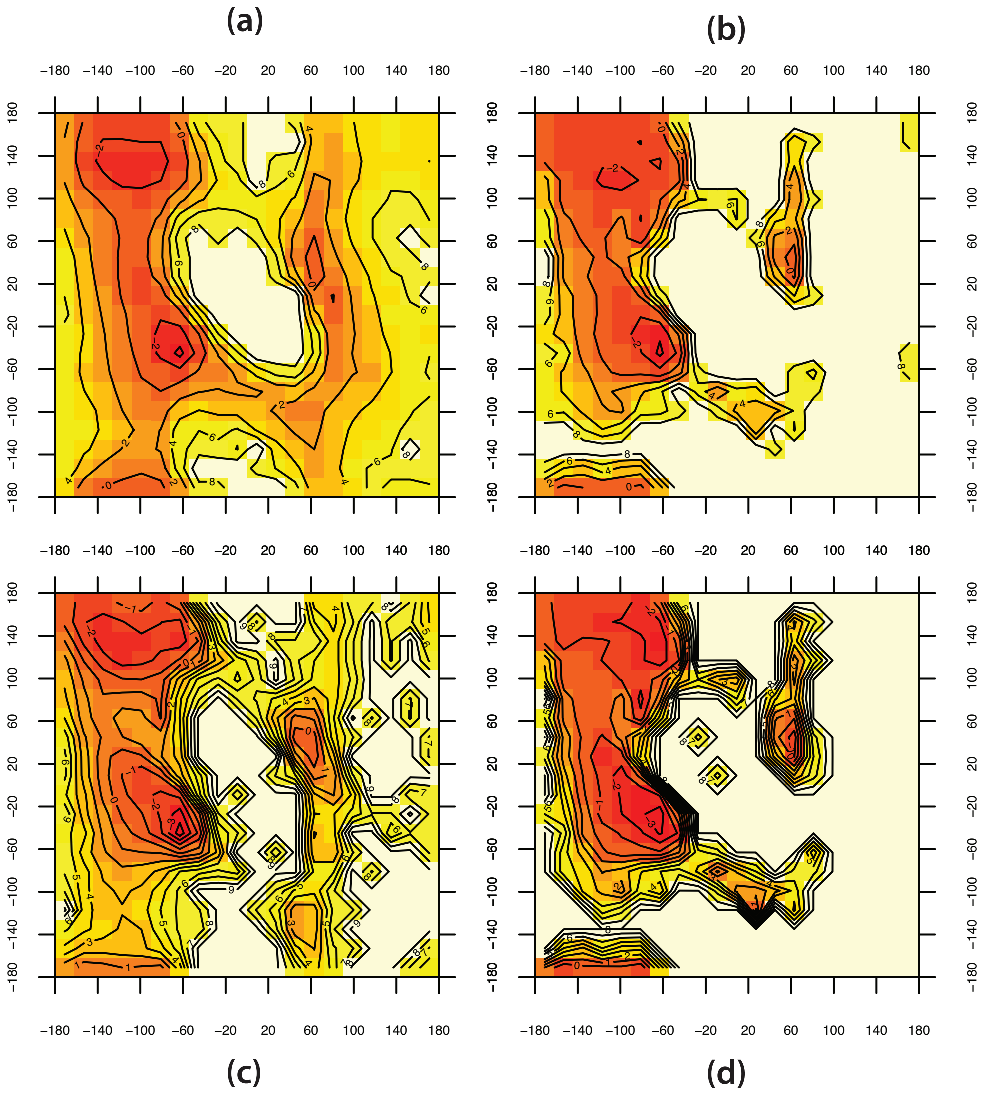

Figure S1: Log likelihood Ramachandran distributions of (a) the novel scaffold decoys ("decoys") before Rosetta design/relax, (b) the novel scaffold decoys ("decoys") after Rosetta design/relax, (c) the high resolution PDB training set, and (d) the compact random coil controls ("random") after Rosetta design/relax.

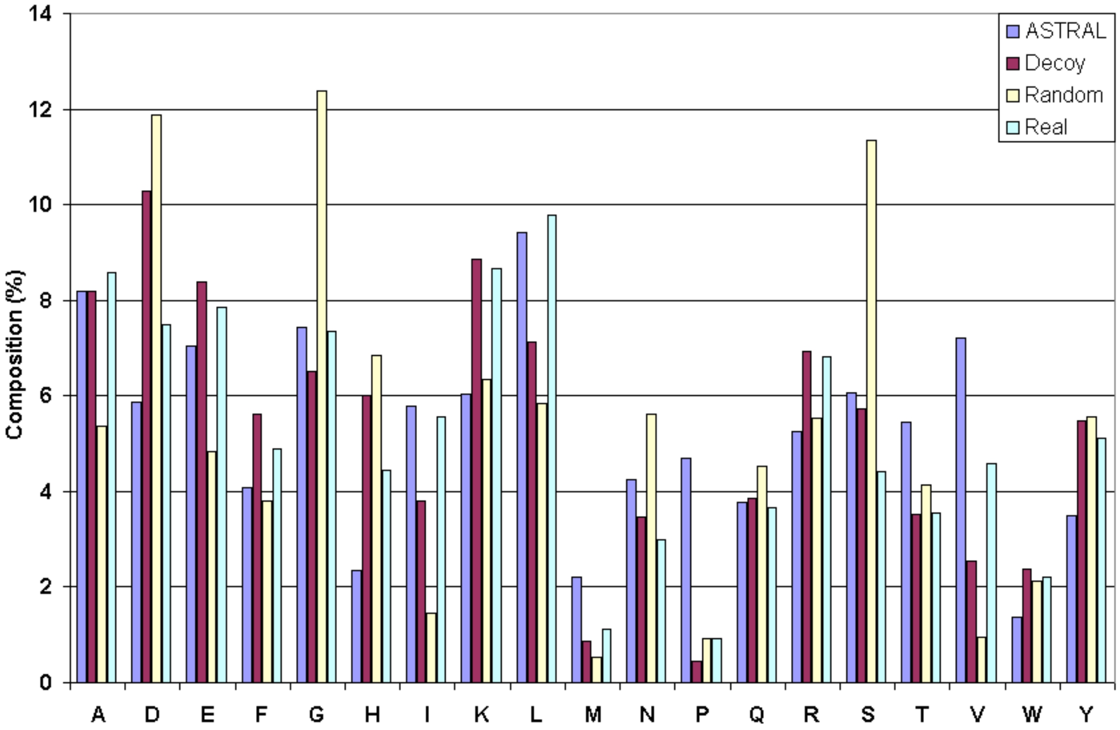

Figure S2: Overall residue compositions for sequences from original design protocol

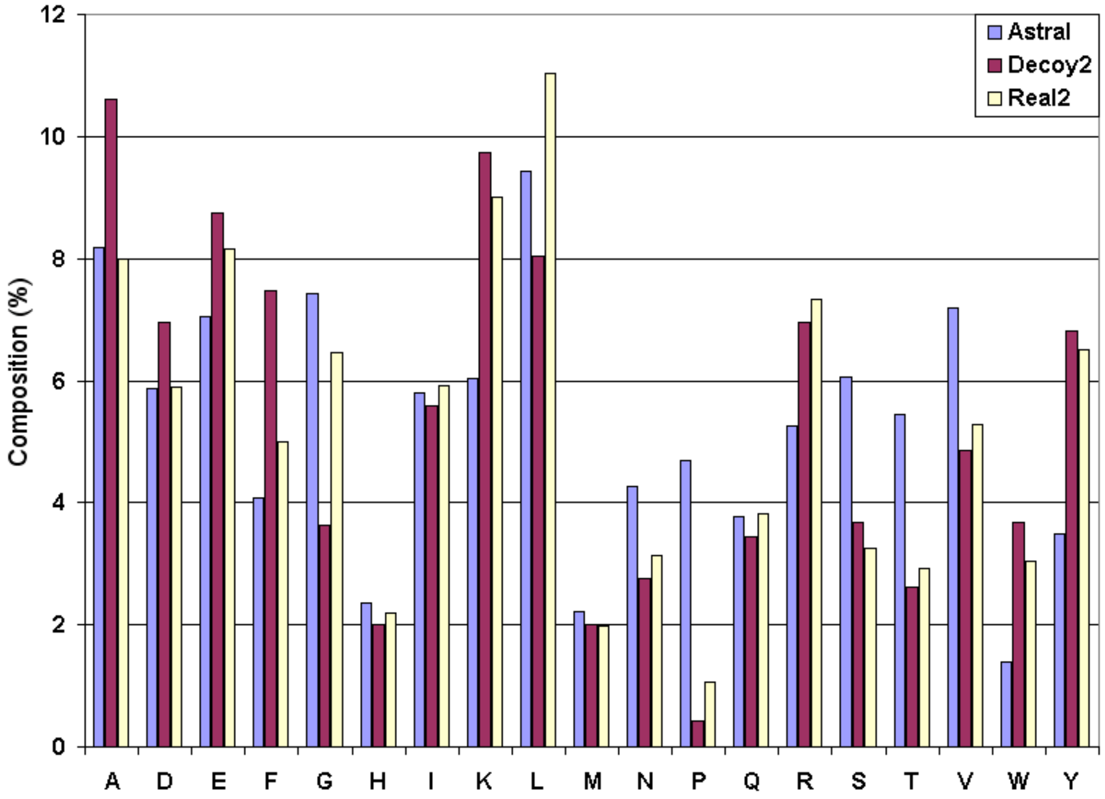

Figure S3: Overall residue compositions from modified protocol

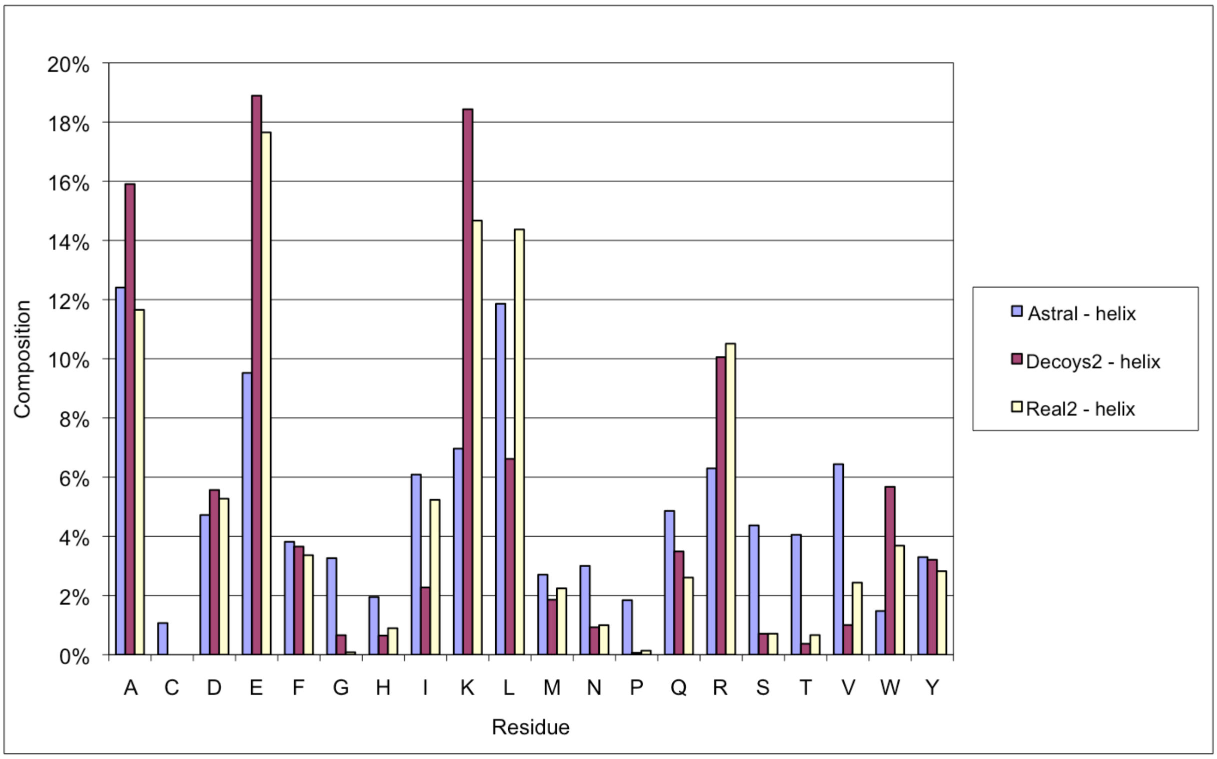

Figure S4: Residue compositions in helical secondary structure

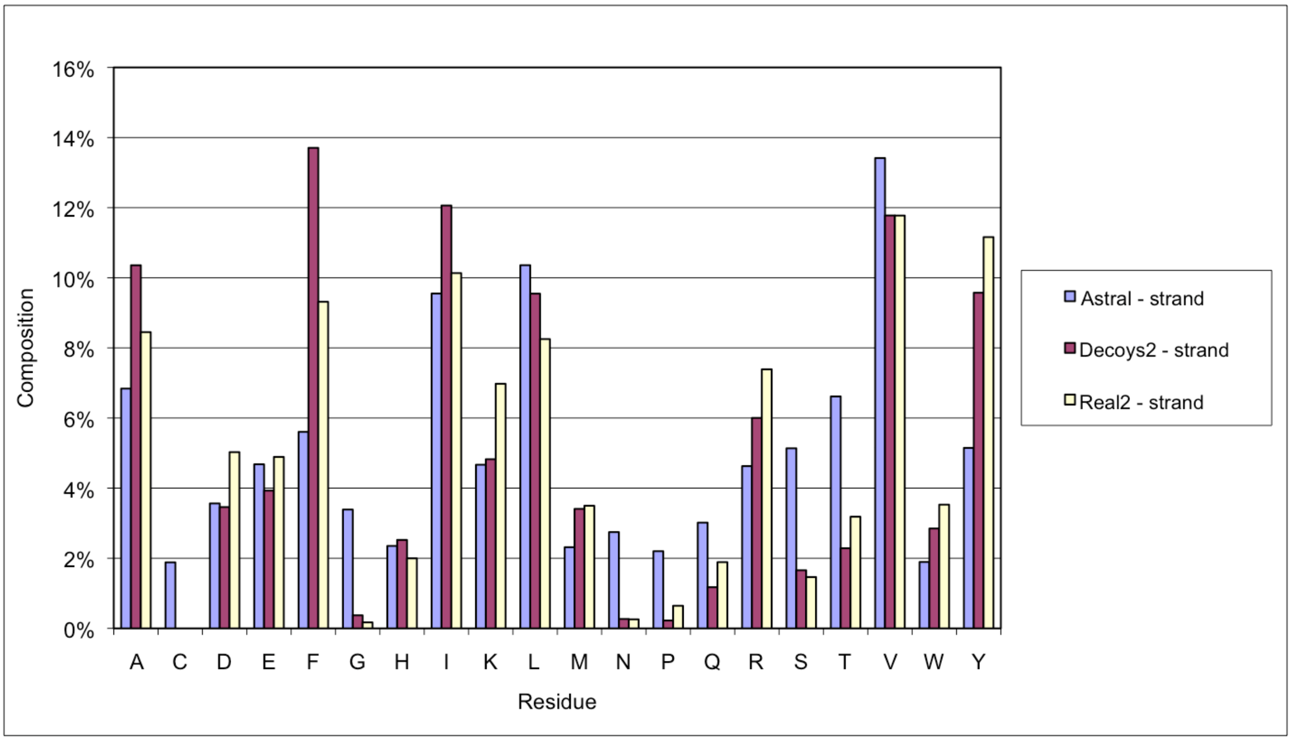

Figure S5: Residue compositions in beta-strand secondary structure
